# Assessing the contribution of prescribing in primary care by nurses and professionals allied to medicine: a systematic review of literature

**DOI:** 10.1186/1472-6963-11-330

**Published:** 2011-12-02

**Authors:** Sadiq Bhanbhro, Vari M Drennan, Robert Grant, Ruth Harris

**Affiliations:** 1 Faculty of Health & Social Care Sciences, St. George's University of London & Kingston University, Grosvenor Wing, Cranmer Terrace, London, SW17 ORE, UK

## Abstract

**Background:**

Safe and timely access to effective and appropriate medication through primary care settings is a major concern for all countries addressing both acute and chronic disease burdens. Legislation for nurses and other professionals allied to medicine to prescribe exists in a minority of countries, with more considering introducing legislation. Although there is variation in the range of medicines permitted to be prescribed, questions remain as to the contribution prescribing by nurses and professionals allied to medicine makes to the care of patients in primary care and what is the evidence on which clinicians, commissioners of services and policy makers can consider this innovation.

**Methods:**

A integrative review of literature on non-medical prescribing in primary care was undertaken guided by dimensions of health care quality: effectiveness, acceptability, efficiency and access.

**Results:**

19 papers of 17 empirical studies were identified which provided evidence of patient outcome of non medical prescribing in primary care settings. The majority were undertaken in the UK with only one each from the USA, Canada, Botswana and Zimbabwe. Only two studies investigated clinical outcomes of non-medical prescribing. Seven papers reported on qualitative designs and four of these had fewer than ten participants. Most studies reported that non medical prescribing was widely accepted and viewed positively by patients and professionals.

**Conclusions:**

Primary health care is the setting where timely access to safe and appropriate medicines is most critical for the well-being of any population. The gradual growth over time of legislative authority and in the numbers of non-medical prescribers, particularly nurses, in some countries suggests that the acceptability of non-medical prescribing is based on the perceived value to the health care system as a whole. Our review suggests that there are substantial gaps in the knowledge base to help evidence based policy making in this arena. We suggest that future studies of non-medical prescribing in primary care focus on the broad range of patient and health service outcomes and include economic dimensions.

## Background

Safe and timely access to effective and appropriate medication through primary care settings is a major concern for all countries addressing both acute and chronic disease burdens [1]. From the nineteenth century onwards, governments have responded to concerns for public protection and concerns about drug misuse through medicine regulation legislation. By the twentieth century, legislation started to incorporate prescriptive authority restricted to a small number of occupational groups such as doctors, dentists and vets for certain classes of drugs [2]. Medicine regulation has developed at different rates in high and low income countries, as has the mechanisms to enforce them. The development in the later part of the twentieth century of a more effective range of medicines has seen a different set of public health preoccupations which range from the prevention of antibiotic resistance, to issues both of how to fund and contain medicine costs in health care systems and at the same time ensure equity of access for citizens to basic health care and essential medicines [1]. Each country places different emphasis on these issues but in many it has led to consideration of the use of other health professional groups in addition to doctors to prescribe regulated classes of medicines or medicines provided through a state sponsored or funded health care provision.

Non-medical prescribing (NMP) is one term that is used to describe the extension of prescriptive authority to professional groups other than the medical profession such as nurses, midwives and allied health professions. Data is not easily available on the extent of NMP for all 194 member states of the World Health Organisation. We have identified, through internet searches, the literature search described below and personal communications, 22 countries which have legislation giving prescriptive authority to nurses (see Table 1[3,4]).

**Table 1:** Countries with identified legislation for Prescribing by Nurses

Country	Legal Framework for Non-medical prescribing (NMP)
Australia	The Drugs Poisons and Controlled Substances Act 1981 and Nurses Act 1993 (Vic) amended with Nurse Amendment Act 2000*.

Botswana	The Drugs and Related Substances Act 1992 grants legal authority to RGN to prescribe specific drugs from Botswana National Drug Formulary***.

Canada	The College of Registered Nurses of Nova Scotia Regulations (Section 27, 2002), approved by government, enabled NPs to prescribe from a restricted list of medications known as the Authorized Practices Schedule*.

Kenya	Public Health Act Cap 242 of the laws of Kenya allows nurses to diagnose and treat minor illnesses in settings without doctors *.

New Zealand	A 2001 regulation under the Medicines Act 1981 enables nurse practitioners to become designated prescribers within their defined area of practice (e.g., primary health)*.

Namibia	Under Nursing Act (8) 2004 in Namibia Nurse Prescribers are allowed to prescribe up to Schedule 4 medicines once they have concluded a primary care training course from the Department of Health***.

Republic of Ireland	Introduced in 2007. The Irish Medicines Board (Miscellaneous Provisions) Act 2006 (No. 3 of 2006) and its associated regulations and the Nurses Rules 2007 [3].

South Africa	The Section 38A of the Nursing Act, 1978 give rights to nurses to prescribe certain classes of medicine. The 38A section of Nursing Act no longer exists and Act 101 of the Medicines and Related Control Act 1965 amended in 1997 allows to nurses to become an authorised prescriber*.

Sweden	Introduced in 1994. District Nurses may prescribe from a National Board of Health and Welfare *.

Uganda	National Drug Policy and Authority Statute 1993 was amended in 2004, which allows nurses to prescribe***.

United Kingdom (UK)	The Medicinal Products: Prescription by Nurses etc Act 1992, The Health and Social Care Act 2001**.

United States of America (USA)	Nurse Prescribing (NP) introduced in 1969. Fifty States allow some form of NMP. However, there is no uniformity in law, language and regulations among States*.

In addition we are aware that NPM legislation exists in Cameroon, Zimbabwe, Rwanda, Swaziland, Malawi, Tanzania, Zambia, Ghana, Lesotho and Ethiopia****, but were unable to identify the specific legislative documents.	

*International Council of Nursing 2009 [4] ** Department of Health *** Personal communication **** Internet search

Some other countries, for example Spain, the Netherlands, Finland, Jamaica and Hong Kong have initiated efforts to introduce legalisation on NMP [4]. There is great variety in the prescribing legislation of different countries. Some countries legislate for the initial qualification and registration of the nurse as sufficient to undertake prescribing certain classes of medicines and in certain situations, for example Kenya, while others require further qualifications, for example Namibia. Within a country there can also be variations between the extent of classes of medicines nurses can prescribe either as a result of different state legislation, for example the United States of America (USA) or different levels of prescribing qualifications, for example in the United Kingdom (UK). In the UK 'independent prescribing' qualifications allows almost all medicines to be prescribed within the individual's clinical competence and 'community practitioner prescribing' qualification gives only authority over a small limited nurses formulary. In addition, many countries have mechanisms whereby individual nurses (or other professionals) have authority, agreed by their employer and/or doctor responsible for a service, to prescribe and dispense or administer a specified list of medicines to a pre-defined group of patients in specific circumstances and within specified parameters. Common international examples of this are within public health immunisation programmes [5]. These are known by a variety of names such as standing orders. In the UK these are known as patient group directions [6] and are used widely across the spectrum of health services [7].

In the last fifty years different models of primary care have developed in countries, influenced by health care funding, government policies and the aspirations of family medicine and general practice [8]. The extent to which groups of professionals such as nurses and pharmacists are present in the primary care system of each country is dependent on both history and these current policy imperatives. This varies between countries even neighbouring in the same continent, for example, the UK has seen significant numbers of nurses employed in general practice over the past thirty years [9], where as there are small numbers of nurses employed in primary care in France [10].

Extending prescribing authority in primary care is a health care innovation driven by various factors in each country. Addressing shortages of medical staff particularly in remote and rural areas has been one driving factor in North America, Africa and Australia [11,12]. In African countries such as South Africa, Botswana, Uganda and Zimbabwe, the aim has been to meet community health care needs by improving access to medicines [13]. In Sweden, the UK and New Zealand; NMP was commenced in order to improve the efficiency of services for specific groups, such as elderly people or those who receive nursing care in the community [14,15]. In some countries, the aspirations of professional groups have been significant in changes to the legislation [16] but only when they have coincided with other public health and health policy imperatives. In summary, the key policy goals to date have been to improve patient access in primary care settings to safe, timely and effective medicines and increasing the efficiency of health service delivery. However, NMP exists in a minority of countries and the extent of prescriptive authority is contentious in some [17]. A sociological narrative review has explored these dimensions further [18]. Other recent narrative reviews [19,20] have considered nurse prescribing in any setting without acknowledging that prescribing in primary care is a very different context from a hospital setting. In primary care settings the prescriber may have little immediate access to other professionals and may be seeing patients with previously undiagnosed illnesses. Therefore the question remains as to the contribution NMP makes to the care of patients in primary care and what is the evidence on which clinicians, commissioners of services and policy makers can consider this innovation.

There is increasing interest in many health care systems to evaluate interventions and innovations in terms of the outcomes for patients, rather than just examine structural and process elements. Donabedian defines the outcome of care as "the effects of care on the health status of patients and populations. Improvements in the patient's knowledge and salutary changes in the patient's behaviour are included under a broad definition of health status, and so is the degree of the patient's satisfaction with care" [21], p. 1745. Donabedian differentiates this from the structural elements i.e. the attributes of the setting in which care occurs and the process elements i.e. what is actually done in giving and receiving care [21]. This paper reports on an integrative review of the empirical literature [22] which addressed the question what is the effect of NMP in primary care and community settings on patient outcomes?

## Methods

A search strategy was devised to include published and grey literature. The electronic data bases CINAHL, MEDLINE, BNI, AMED, ISI Web of Knowledge and Index to theses were searched. A search to retrieve grey literature was also conducted of relevant websites: Google scholar, the Royal College of Nursing, Royal Pharmaceutical Society, NHS Modernisation Agency, King's Fund, National Institute of Clinical Excellence, Department of Health, and National Prescribing Centre. Searches also included follow up of reference lists and key authors. Searching was conducted by SB according to the inclusion and exclusion criteria, using terms of NMP.

The search terms employed were nurse prescribing, non-medical prescribing, supplementary prescribing, independent prescribing, pharmacist prescribing, allied health professional prescribing, prescribing rights and prescribing impact and outcomes. A combination of these search terms was used. All items within each terms section were combined with OR and then each section was combined with AND for different combinations of sections that produced the highest result. The inclusion criteria and the exclusion criteria were as follows:

### Inclusion criteria

• Study contains empirical evidence of NMP from any professional group with legislative authority

• Study contains empirical evidence of outcomes

• Setting: primary care and community

• Search period: January 1970 - December 2010 for the USA and October 1994 - December 2010 for the UK and other countries. These timeframes reflect the years when non-medical prescribing was introduced in these countries.

### Exclusion criteria

• Studies that did not meet inclusion criteria above

• Commentaries, editorials, opinions, guidelines and service audits

• Papers that did not report the research design or methods used

Abstracts were identified, screened by two researchers and accepted or rejected based on the inclusion and exclusion criteria. Full papers were obtained where the abstract was unclear to enable an accurate decision. Full papers were obtained for all included papers and data on sample size, prescribing authority type, findings on outcomes and process outcomes (as defined above) and study limitations were extracted. Each study was considered against the adapted quality checklists relevant to the study design [23,24]. Regular meetings were held between the researchers to discuss and agree interpretations and to clarify any inconsistencies in the evidence.

Due to the heterogeneity of study methods, participants and outcomes, an overall meta-analysis was not appropriate. Instead data are presented narratively through a synthesis framed by the dimensions of judging health care suggested by Maxwell [25]. These dimensions are of the effectiveness, acceptability, efficiency, equity (fairness) and access to health care. The definitions as described by Maxwell were used. Effectiveness addresses questions of "whether the treatment or intervention is the best available in the technical sense and the overall result of the treatment" [25], p. 171. Acceptability considers questions of patient's perceptions. Efficiency considers questions of "whether the output is maximised for the given input or conversely whether the input is minimised to achieve the stated output" [25], p. 171. Finally, access considers the questions of whether people receive treatment/service when they need it [25].

## Results

The initial searches produced 1734 abstracts, of which 961 duplicate articles were removed. Titles and abstracts were screened by SB and VMD. After reading titles and abstracts 375 of the 773 papers were excluded. The remaining 398 papers were classified into three categories: empirical papers (n = 184), opinion papers (n = 209) and literature reviews (n = 5). The opinion papers and reviews were excluded.

**Figure 1:**
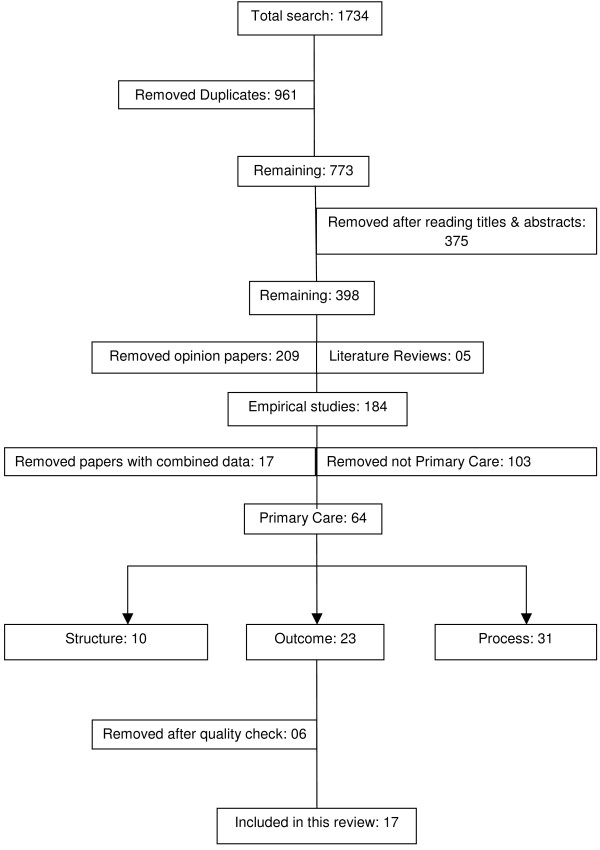
**Flow chart of the selection of papers in review**.

One hundred and three of the 184 empirical papers were excluded as they did not relate to primary care settings and 17 were excluded as they reported combined primary and acute care data and it was not possible to separate the data relating to primary care (see table 2[26-41]). The full text of the remaining 64 papers were read (SB, RG, RH and VMD) and categorised as to whether they addressed questions of structure, process, or outcomes of NMP in primary care. Those presenting evidence only on the structure or process were excluded (n = 41). In six of the twenty-three remaining papers the research question and/or method were unclear or omitted important information to enable the quality of the paper to be assessed and were therefore excluded. A total of 19 articles of 17 studies reporting on the outcomes of NMP were included in the review (Figure 1). Two studies used the same data for two publications. Details of the papers are given in Additional File 1. Most studies were conducted in the UK. The majority investigated the contribution of nurses as a non-medical prescriber with a small number investigating the more recent development of pharmacists as non-medical prescriber (Table 3). Of the 17 studies, seven used qualitative methods only, eight quantitative methods and two employed mixed methods designs. We now turn to consider the evidence within the studies as grouped by questions of effectiveness, efficiency, acceptability and access. Issues of equity are considered within the section on access.

**Table 2:** Papers and reports with combined primary care and secondary care data

Complete reference	Country
Latter S, Blenkinsopp, A., Smith, A., Chapman, S., Tinelli, M., Gerard, K., Little, P., Celino, N., Granby, T., Nicholls, P., Dorer, G.: **Evaluation of nurse and pharmacist independent prescribing**. London: University of Southampton & Keele University; 2010 [26].	UK

Hoti K, Sunderland B, Hughes J, Parsons R: **An evaluation of Australian pharmacist's attitudes on expanding their prescribing role**. *Pharmacy World & Science: PWS *2010, **32**(5):610-621 [27].	Australia

Hacking S, and Taylor, J: **An evaluation of the scope and practice of non-medical prescribing in the North West for NHS North West**. Lancashire: School of Nursing & Caring Sciences, University of Central Lancashire 2010 [28].	UK

Hobson RJ, Scott J, Sutton J: **Pharmacists and nurses as independent prescribers: exploring the patient's perspective**. *Family Practice *2009, **27**(1):110-120 [29].	UK

Drennan J. NC, Allen D., Hyde A., Felle P., O'Boyle K., Treacy P., Butler M.: **Independent Evaluation of the Nurse and Midwife Prescribing Initiative**. Dublin: University College Dublin; 2009 [3].	Republic of Ireland

Courtenay M, Stenner K, Carey N: **An exploration of the practices of nurse prescribers who care for people with diabetes: a case study**. *J Nursing & Healthcare of Chronic Illness *2009, **1**(4):311-320 [30].	UK

Dunn SV, Cashin A, Buckley T, Newman C: **Nurse practitioner prescribing practice in Australia**. *Journal of the American Academy of Nurse Practitioners *2008, **22**(3):150-155 [31].	Australia

Courtenay M, Carey N: **Nurse independent prescribing and nurse supplementary prescribing practice: national survey**. *Journal of Advanced Nursing *2008, **61**(3):291-299 [32].	UK

Courtenay M, Carey N: **Preparing nurses to prescribe medicines for patients with diabetes: a national questionnaire survey**. *Journal of Advanced Nursing *2008, **61**(4):403-412 [33].	UK

Cooper R, Anderson C, Avery T, Bissell P, Guillaume L, Hutchinson A, Lymn J, Murphy E, Ratcliffe J, Ward P: **Stakeholders' views of UK nurse and pharmacist supplementary prescribing**. *Journal of Health Services Research & Policy *2008, **13**(4):215-221 [34].	UK

Bissell P, Cooper, R., Guillaume, L., Anderson, C., Avery, A., Hutchinson, A., James, V., Lymn, J., Marsden, E., Murphy, E., Ratcliffe, J., Ward, P., and Woolsey, I: **An Evaluation of Supplementary Prescribing in Nursing and Pharmacy**. London: Department of Health 2008 [35].	UK

Latter S, Maben J, Myall M, Young A, Baileff A: **Focus. Evaluating prescribing competencies and standards used in nurse independent prescribers' prescribing consultations: an observation study of practice in England**. *Journal of Research in Nursing *2007, **12**(1):7-28 [36].	UK

Latter S, Maben J, Myall M, Young A: **Evaluating the clinical appropriateness of nurses' prescribing practice: method development and findings from an expert panel analysis**. *Quality & Safety in Health Care *2007, **16**(6):415-421 [37].	UK

Courtenay M, Carey N, Burke J: **Independent extended and supplementary nurse prescribing practice in the UK: a national questionnaire survey**. *International Journal Of Nursing Studies *2007, **44**(7):1093-1101 [38].	UK

George J, McCaig DJ, Bond CM, Cunningham ITS, Diack HL, Watson AM, Stewart DC: **Supplementary prescribing: early experiences of pharmacists in Great Britain**. *The Annals Of Pharmacotherapy *2006, **40**(10):1843-1850 [39].	UK

Flenniken MC: **Psychotropic prescriptive patterns among nurse practitioners in nonpsychiatric settings**. *Journal of the American Academy of Nurse Practitioners *1997, **9**(3):117-121 [40].	USA

Batey MV, Holland JM: **Prescribing practices among nurse practitioners in adult and family health**. *American Journal of Public Health *1985, **75**(3):258-262 [41].	USA

**Table 3:** Distribution of papers by country and type of NMP

Country	No. Of papers	Nurses	Pharmacists
UK	11	X	
	
	02		X

USA	01	X	

Canada	01	X	

Botswana	01	X	

Zimbabwe	01	X	

### Effectiveness

Effectiveness addresses questions of whether the treatment or intervention is the best available in the technical sense and the overall result of the treatment [25]. Fifteen of the seventeen studies investigated some aspect of the effectiveness of NMP in primary care. Of these 15 studies, thirteen investigated nurse prescribing and two pharmacists prescribing. Six of these studies used qualitative semi-structured and in-depth interviews [42-47], seven used quantitative questionnaire surveys and secondary data analysis [48-54] and two applied a mixed methods approach [55,56]. The majority of studies considered effectiveness of service delivery and only one of the studies considered therapeutic effectiveness [53].

Four studies from UK, Canada, Botswana and Zimbabwe, which analysed patient clinical accounts, reported substantial increases in the prescription by NMP of non-steroidal anti-inflammatory [49], cardiovascular [50] and antibiotic medicines [54-56]. They give an indication of both the conditions that non-medical prescribers were encountering and also of the numbers or confidence of non-medical prescribers to prescribe these medicines. Only two studies presented comparative data from general practitioners indicating similar frequency of prescribing but not by groups of medicines [51,53].

Three studies describing patient views [[43,48] and [57]] and one of clinical consultation review [58] reported that NMP were effective in improving the provision of information, advice and understanding on treatment, conditions, self-care and standard of care. One study describing data from the professional viewpoints reported that NMP had enhanced concordance with patients [46]. One study which analysed clinical records [53] reported that the NMP intervention improved patient reported outcomes of treatment. None of these studies present any other data as evidence to support these viewpoints.

Most of the studies were not able to comment on the effectiveness of NMP in primary care in relation to safety and appropriateness of prescribing by nurses and other professionals allied to medicine. However, there is some evidence from two studies in two different African countries that some prescribing of antibiotics by those nurses may have been inappropriate and not evidence-based [54,56]. Neither presents comparative data to judge whether other professionals such as doctors in that type of setting prescribed in similar or different ways. A mixed methods survey of adherence to treatment guidelines in primary health care facilities in Botswana found that antibiotics were prescribed in 27% of all 2994 consultations. The study reported that full adherence to prescribing guidelines (defined as complete adherence to national recommended treatment guidelines) occurred in 44% of prescriptions, acceptable compliance in 20%, acceptable but with one or more useless although not dangerous drugs in 33% and insufficient or dangerous treatment in 3% of the consultations [56]. An unspecified survey of antibiotic prescribing by nurses in primary care clinics in Harare, Zimbabwe found that of 1000 patient (presumably records but not specified in the paper) surveyed 543 were prescribed with antibiotics. It was reported that 12.3% of patients were prescribed antibiotics inappropriately [54]. The same study referenced an unpublished paper reporting on a previous small survey which was carried out in a paediatric primary care clinic in Zimbabwe. The study reported that 55% of children were treated with antibiotics when seen by the nurses but only 22% when seen by a paediatrician [59].

### Efficiency

Efficiency considers questions of whether the output is maximised for the given input or conversely whether the input is minimised to achieve the stated output [25]. Of the 17 studies nine considered questions of efficiency; seven of them as a part of a broader study and two considered only questions of efficiency using qualitative [42,45] and quantitative [58] methods. All these studies reported prescribing by nurses.

Four studies, one of patient [42] and three of professional views [[44,47] and [55]], reported that NMP was efficient in that it was viewed as easy, convenient and timely without the need to wait for a GP appointment. Two studies of nurses' views reported that being able to prescribe enabled them to provide seamless and patient centred care [45,46]. A UK based observational survey of clinical accounts reported that nurse prescribers fully completed the episode of care, i.e. did not have to refer on to a doctor, in 65% of patients presenting in the same day appointments using a combination of advice and prescriptions [58]. In an American evaluation of Advanced Nursing Practitioners (ANP) with prescriptive authority 1,708 patients were seen and prescribed by 32 ANPs. An analysis of patient records found that patients experienced short waiting times (63% waited 15 minutes or less) [53]. A Canadian study which analysed two years of prescription claims by older adults reported that the number of prescriptions per nurse prescriber doubled and cost per prescription increased approximately 20% over the time period [49]. The authors noted the increase in cost per prescription; however, they did not interpret this in terms of efficiency. While three studies describing the professional views [[44,49] and [55]] reported that NMP was time saving for patient and nurses only one study, which was conducted in the UK, reported NMP as a cost-effective intervention [47].

### Acceptability

Acceptability considers questions of suitability and satisfaction from the perspective of both those receiving the intervention (the patients) and others providing or commissioning the service (the professionals and managers) [25]. It therefore relates to perceptions of outcomes. Of the 17 studies in the review, three reported views about acceptability as a part of a broader study. Of these three studies, two investigated nurse prescribing and one pharmacist prescribing. The studies found that NMP was widely accepted and viewed positively by patients [[42,43,48] and [60]]. A UK based qualitative study that interviewed 50 patients from caseloads of health visitors (n = 17), district nurses (n = 9) and a practice nurse (n = 1) reported that 49 (98%) out of 50 study participants were in favour of nurse prescribing and happy with the consultation and information provided by the nurse prescribers [42]. Similarly, another UK based study interviewed a sample of 148 patients selected from the caseloads of district nurses, health visitors and practice nurses after the treatment episode involving non-medical prescriber. The majority of patients interviewed post prescribing implementation, were in favour of nurse prescribing and 55% of patients interviewed had sought advice from a nurse prescriber in preference to the GP [43].

### Access

Access considers the questions of whether people receive a treatment or service when they need it and whether there are any identifiable barriers to service uptake [25]. Five studies considered the question of access as a part of a broader study. Four studies considered nurse prescribing and one pharmacist prescribing from UK. Four patient views studies [[42,43,48] and [61]] and one clinical consultation review analysis [58] reported that introduction of NMP has improved access to medicines and health care professionals. A UK based qualitative study, which interviewed 41 patients from caseloads of seven nurse prescribers, reported that they thought that their access to medicine had improved during non-routine/non-emergency appointments [61]. Similarly, another UK study interviewed 305 patients selected from the caseloads of nurse prescribers reported that patients appreciated the nurses being accessible resulting in no delay in starting medication [43]. A questionnaire study investigating the patients' experience (n = 127) of pharmacist-led supplementary prescribers in a UK primary care setting reported that 86% of respondents stated that they are able to make appointments easily, which resulted in improved access to medicines [48].

## Discussion

While there have been previous published reviews of non-medical prescribing, none have considered the evidence from one setting, in this case primary care, or have focused on outcomes.

In this review, 19 papers of 17 empirical studies (two studies published two articles each) were identified which provided evidence of patient outcome of NMP in primary care settings. The majority were undertaken in the UK with only one each from the USA, Canada, Botswana and Zimbabwe. Seven papers report on UK studies of nurse prescribing from a limited nurses' formulary. Seven papers reported on qualitative designs and four of these had fewer than ten participants. Two reported on surveys of opinion and experience. Eight papers reported on record reviews of prescriptions or clinical consultation by NMPs. Those studies that provide objective measures are mainly descriptive. Only one provided some comparative evidence of another type of prescriber, GPs, by which to judge the impact on patient outcomes or outcomes on the efficiency for the health system [52]. While there may be a publication bias in reporting positive outcomes present in those identified, many of the studies included in the review had design weaknesses and limitations, both as indicated by the authors and evident through critical appraisal of the papers. The strength of evidence they provide on the whole is limited.

The review findings from stakeholders' perspectives suggest that NMP in primary care effectively improves patients' understanding of treatment, condition and self-care and provides a better level of care. As the literature suggests that concordance is a major issue in the effective use of medicines in primary care settings [62] the impact of additional information and advice may be significant in considering which type of prescriber is effective for which particular patient groups. This proposition requires further testing and investigation.

We found very limited evidence in relation to other indicators of effectiveness of NMP outcomes such as of patient safety and clinical outcomes. The overall number of research-based studies to evaluate impact and outcome of NMP was low given that NMP was introduced in many countries over 30 years ago. In part this reflected the number of papers excluded as it was not possible to separate primary care related data from secondary care related data but it may also be that NMP is seen as producing positive outcomes in situations where there are no alternative prescribers. This may explain the absence of empirical outcome evidence from low income countries in particular, although this may also reflect the review search strategy, which did not search country specific journals not indexed on the major electronic databases. Given that it is a minority of countries that have given prescribing authority to professionals other than doctors and dentists, it may be that it is this type of evidence that would be of value to policy makers and requires further investigation and publication.

In relation to efficiency of NMP in primary care, the review suggests that patients received services that were timely, seamless and of high quality from nurse and pharmacist prescribers. One study reported opinions that NMP was cost effective in primary care. We were unable to find any papers from a health economics perspective or that modelled the efficiency impact from either patient or the health services perspective. We suggest that this is an aspect that warrants further investigation.

All the studies investigating acceptability of NMP indicated that NMP was well accepted and favoured by patients, nurses, pharmacists and other health care professionals. The gradual growth over time of legislative authority to NMP and also of the numbers of non-medical prescribers, particularly nurses, in countries such as the USA and the UK, suggests that the acceptability is based not just on immediate levels of satisfaction with the clinical encounter but perceived value to the health care system as a whole.

The review findings also report that patients considered it was easier, quicker and convenient to get an appointment with NMP and their access to medicine and health care professionals was improved. For all countries the issue of timely access to appropriate medicines has health service and public health ramifications. For countries with well developed primary care services such as the UK, the ability of primary care professionals other than doctors to provide consultations that include prescribing may improve waiting times to consult and help manage demand and potential dissatisfaction. The issue of equitable access to safe and affordable medicine is critical for lower-income countries where the access to medicines is compromised by insufficient health facilities and staff, low investment in health and the high cost of medicines [63]. In these settings if legislative authority to prescribe is not extended to groups other than doctors and dentists, using mechanisms such as patient group directions or standing orders for community health workers for a specified essential drug list and immunisation list may have significant and critical public health impact. The contribution of these types of mechanisms with a broader group of community health staff is not within the scope of this review but warrants further investigation.

This review has limitations in that it included only English language studies and those accessed through electronic sources and therefore may have excluded evidence from many Scandinavian, African, South East Asian and South American countries. However, our review of countries that have legislated for prescribing authority for professionals other than doctors and dentists would suggest that researchers from many of these countries are likely to publish evidence in English language journals, although not necessarily ones that are indexed through the databases we searched.

Our focus on patient and health service outcomes has been both a strength and a weakness: while outcomes are important, the small number of studies finally included demonstrate how limited the evidence is. We argue that it is these aspects that most urgently need investigation. Our focus on solely primary care has also meant that we have had to exclude some more recent studies providing evidence from mixed primary and secondary care settings, aspects such as clinical appropriateness of NMP, e.g. Drennan et al, 2009, Latter et al, 2010, and Bissell et al, 2008 [3,26,35]. In many of these studies there were substantial numbers of NMP in primary care settings. We suggest that secondary data analysis of some of these studies by health care setting may be invaluable to providing evidence for service planners, commissioners and managers.

## Conclusions

NMP has been implemented and evolved differently in different countries. Around twenty countries out of 193 member states of the World Health Organisation (WHO) provide legal authority to nurses and other professionals allied to medicine to prescribe medicines at a certain level and others are considering introducing legislation. This suggests that internationally twenty first century policy makers are beginning to look as to how to move beyond twentieth century established professional boundaries for the benefit of both public health and their health care economy. Primary health care is the setting where timely, and equitable, access to safe and appropriate medicines is most critical for the well-being of any population. Our review suggests that there are substantial gaps in the knowledge base to help evidence based policy making in this arena. We suggest that this review indicates there is a need for secondary data analysis of existing studies and commissioning of new studies that address questions of non-medical prescribing in primary care across a broad range of patient and health service outcomes, including economic dimensions.

## Competing interests

The authors declare that they have no competing interests.

## Authors' contributions

VMD conceived the study. All designed the study. SB undertook the search and initial reading. All undertook second reading and quality checks. SB produced the first draft of the paper and all contributed to revisions. All authors read and approved the final manuscript.

## Pre-publication history

The pre-publication history for this paper can be accessed here:

http://www.biomedcentral.com/1472-6963/11/330/prepub

## Supplementary Material

Additional file 1 **Non-medical prescribing outcomes based papers grouped by patient views, professional views and clinical accounts**. Details of the papers included in the review. Click here for file
