# Supplementary material for: Assessing the contribution of prescribing in primary care by nurses and professionals allied to medicine: a systematic review of literature
Source: BMC Health Serv Res. 2011 Dec 2;11:330. doi: 10.1186/1472-6963-11-330 (PMC3248914; doi:10.1186/1472-6963-11-330)
Supplement: Additional file 1 — Non-medical prescribing outcomes based papers grouped by patient views, professional views and clinical accounts. Details of the papers included in the review. [file 1472-6963-11-330-S1.DOC]

## Non-medical prescribing outcomes based papers grouped by patient views, professional views and clinical accounts

| **Study Reference**  **(ordered by year)** | **Method/sample** | **Prescribing Type** | **Results** | **Stated Limitations** | **Appraisal notes (regarding clarity of questions & methods)** |
| --- | --- | --- | --- | --- | --- |
| **Patients views, opinions, perceptions and experiences** | | | | | |
| Stenner KL, Courtenay M, Carey N: Consultations between nurse prescribers and patients with diabetes in primary care: A qualitative study of patient views. *International Journal Of Nursing Studies* 2010, 48(1):37-46 [61].  Courtenay M, Stenner K, Carey N: The views of patients with diabetes about nurse prescribing. *Diabetic Medicine: A Journal Of The British Diabetic Association* 2010, 27(9):1049-1054 [57]. UK | Method: Qualitative semi-structured interviews  Sample: 41 patients selected from case-load of 7 Nurse Prescribers  Analysis: Thematic analysis using ATLAS Ti package. | UK Nurse Independent Prescribing (NIP) | Study found that patient viewed nurse consultation as non-rushed, detailed and flexible; this approach helped them to feel comfortable expressing themselves. Identified nurse prescribing (NP) benefits: person-centred nursing, improved understanding of treatment and condition, improved self-care, better access to medications, access to current advice and treatment, individualised treatment and advice. Second paper concluded that the patients noted that it was easier and quicker to get a non-review appointment with the nurse prescriber, and access to medicines was improved during non-routine/emergency. | As a qualitative study, the findings may not represent the views of all patients.  The study findings could have strengthened if more nurses working in community clinics had been recruited. | Methods and research questions are clearly stated. |
| Smalley L: Patients' experience of pharmacist-led supplementary prescribing in primary care. *The Pharmaceutical Journal* 2006, 276:567-569 [48]. UK | Method: Quantitative self-administrated postal questionnaire  Questions: closed, multi-response  Sample: 127 patients  Response rate: 111/127 (87%) | Pharmacist Supplementary Prescribing | Study found that 57% of respondents stated that the standard of care they received was better than they received previously, 86% of respondents stated that they are able to make appointment easily, which resulted in improved access to medicines and 92% of respondents stated that supplementary prescribing by pharmacists is a good idea. | The non-response rate of 13%, although low, builds a degree of bias into the results. All questionnaires suffer from a degree of recall bias. | Methods and research questions are clearly stated. |
| Brooks N, Otway C, Rashid C, Kilty E, Maggs C: The patient's view: the benefits and limitations of nurse prescribing. *British Journal Of Community Nursing* 2001, 6(7):342-348 [42].  Brooks N, Otway C, Rashid C: Nurse prescribing: what do patients think? *Nursing Standard* 2001, 5(1) [60]. UK | Method: Qualitative face-to-face & telephonic interviews  Sample: 45/50 patients from caseload of Health Visitors (HVs) (n=17), District Nurses (DNs) (n=9) & Practice Nurse (PN) (n=1).  Analysis: Content thematic | UK Nurse prescribing from Nurse Prescribing Formulary (NPF) | The study identified the efficiency and impact of nurse prescribing from patients’ point of view. Majority of patients viewed NP as timely, convenient and practical in terms of information and prescription. Seventy-two percent of the study participants identified timeliness of treatment as a benefit of NP and 46% identified NP as more convenient. Of the 50 participants 49 (98%) were in favour of NP and happy with the consultation and information were provided by the nurse prescribers. Benefits identified were better use of the nurse’s and doctor’s time, convenience, a quality relationship with the nurse and expertise of the nurse. | The study findings may not reflect the views of non-white population from different socio-economic backgrounds.  The participants in this study were mainly low users or new to the experience of nurse prescribing. This did not appear to affect their reflections on current or future service delivery of nurse prescribing. | Methods and research questions are clearly stated. |
| Luker KA, Austin L, Hogg C, Ferguson B, Smith K: Nurse-patient relationships: the context of nurse prescribing. *Journal Of Advanced Nursing* 1998, 28(2):235-242 [43]. UK | Method: Qualitative semi-structured interviews  Sample: convenience sample of 157 & 148 patients selected from the caseloads of DNs, HVs & PNs during pre and post prescribing implementation.  Analysis: thematic content | UK Nurse prescribing from NPF | Study found that patients were in favour of NP and identified benefits of nurse prescribing to include continuity of care, stable nurse-patient relationship, patient, information in relation to prescription received, and nurse being accessible resulting in no delay in starting medication. 55% of patients interviewed had sought advice from a nurse in preference to the GP. | The patients selected for this study were all high users of nurse services and the findings may not be generalised beyond this group. | Methods are quite clear but the research question(s) are not stated, perhaps because the authors write “The purpose of this paper is not to report the patients’ views on prescribing...” |
| **Nurses, pharmacists and other professionals’ views and experiences** | | | | |  |
| Daughtry J, Hayter M: A qualitative study of practice nurses' prescribing experiences. *Practice Nursing* 2010, 21(6):310-314 [44]. UK | Method: Qualitative in-depth interviews  Sample: purposive sample of 8 PN prescribers.  Analysis: Colaizzi’s approach (1978) | UK Unspecified | Study concluded that in participants experience the prescribing not only had time benefits for the nurses, but patients also received effectively and timely services and high quality standards of care from prescribing nurses. | This study only sought to obtain the views of nurses – further views of GPs and other clinical and administrative staff would strengthen and add to the findings of this study. | A clear aim is stated although specific questions in the interview schedule are not reported. Methods are clearly reported. |
| Downer F, Shepherd C: District nurses prescribing as nurse independent prescribers. *British Journal Of Community Nursing* 2010, 15(7):348-352 [45].  UK | Method: Qualitative interviews  Sample: purposive sample of eight DNs.  Analysis: Colaizz’s approach (1978) | UK Nurse Independent Prescribing | Study found district nurses reported benefits of prescribing i.e. time saving, seamless and improved patient care. | Limited generalisability of findings to other areas because this study was undertaken using participants that had attended only one higher education establishment and ultimately across only two health board areas. | A clear objective is stated although specific questions in the interview schedule are not reported. Methods are clearly reported. |
| Davies J: Health visitors' perceptions of nurse prescribing: a qualitative field work study. *Nurse Prescribing* 2005, 3(4):168-172 [46]. UK | Method: Qualitative- semi-structured interviews  Sample: convenience sample of 8 HVs.  Analysis: content analysis | UK Nurse prescribing from NPF | Study reported health visitors perceived patients receive better level of care in many ways with introduction of nurse prescribing. Firstly, they are able to access a service without the need to wait for a GP appointment. Secondly, the patient is a partner in care which from the health visitors’ perspective enhances concordance with patients and thirdly, the patients receive health promotion advice as well as the prescription. | This was very small, used a convenience sample of eight Health Visitors (HVs). | The research question is very broad and not clearly focused. The use of semi-structured qualitative interviews is not clearly reported in terms of questions asked and methods of analysis. |
| Lewis-Evans A, Jester R: Nurse prescribers' experiences of prescribing. *Journal Of Clinical Nursing* 2004, 13(7):796-805 [47]. UK | Method: Qualitative interviews  Sample: purposeful sample of 7 DNs & HVs  Analysis: thematic. | UK Independent prescribing from NPF | Study reported district nurses and health visitors’ perceptions of the outcomes of nurse prescriber to patient centred care – as saving patient time, being convenient, improving communication, increasing patient confidence and continuity of care. | The homogeneity of the sample negates generalisation of the NPs to the population. | Methods are very clearly set out. Research question is very open-ended “experiences of prescribing”, but this is appropriate for a relatively new aspect of health care. |
| Luker K: Nurse prescribing: the views of nurses and other health care professionals. *British Journal of Community Health Nursing* 1997, 2(2):69-74 [55]. UK | Method: Mixed method interviews, focus group discussions and questionnaires  Sample: 58 nurses, community nurse managers, 2 GPs, a practice manager, a Family Health Service Authority (FSHA) pharmaceutical adviser and two local pharmacists. | UK Nurse prescribing from NPF | The study identified the benefits of NP including savings in time for patients and nurses and increased autonomy for nurses. About two-third of nurses interviewed considered that the information provided by nurses at the time a prescription was issued was better than that provided by the GP. This view was supported by 13% of patients and by almost half of the GPs. | None stated | Methods are very clearly set out. Research question is very open-ended, but this is appropriate for the first evaluation of the nurse prescribing “demonstration sites” in the UK. |
| **Data collected from clinical records and consultations** | | | | |  |
| Murphy AL, Martin-Misener R, Cooke C, Sketris I: Administrative claims data analysis of nurse practitioner prescribing for older adults. *Journal Of Advanced Nursing* 2009, 65(10):2077-2087 [49]. Canada | Method: Quantitative prescriptions claims analysis  Sample: 2004/5-2006/7 prescription claims of permanent resident of Nova Scotia with a valid Nova Scotia Health Card and be at least 65 years old. | Canada Nurse Practitioner Prescribing | This analysis found that the prescription volume per nurse doubled and cost per prescription increased by approximately 20% over the time period. Primary health care nurse prescribed antimicrobials and non-steroidal anti-inflammatory drugs consistently represented the top ranked groups for prescription volume and cost. Over the three fiscal years, antimicrobial prescription rates declined relative to rates of other groups of medications. | The results of this study represent a specific time period (i.e. fiscal years 2004/05 to 2006/07) of prescribing. Non-prescription drugs covered by Seniors Pharmacare for any year were not captured. | There is a clear aim but no research question.  Retrospective, population based analysis of prescription claims descriptively analysed |
| Guillaume L, Cooper R, Avery A, Mitchell S, Ward P, Anderson C, Bissell P, Hutchinson A, James V, Lymn J *et al*: Supplementary prescribing by community and primary care pharmacists: an analysis of PACT data, 2004-2006. *Journal of Clinical Pharmacy & Therapeutics* 2008, 33(1):11-16 [50]. UK | Method: Quantitative secondary analysis of Prescription Analysis and Cost (PACT) data at national, chapter and sub-chapter level for 2004-2006. | UK Pharmacists  Supplementary Prescribing | This PACT analysis shows that total number of items prescribed by pharmacists in community & primary care increased from 2706 in 2004 to 31052 in 2006. Cardiovascular medicines were the most frequently prescribed therapeutic class followed by central nervous system, respiratory, endocrine and gastrointestinal medicines. | PACT data offer only a partial picture of pharmacist prescribing. The data analysed were of England only. | The research aim was stated but there were no stated research questions. The method of analysis is not described in detail. |
| Davis K, Drennan V: Evaluating nurse prescribing behaviour using constipation as a case study. *International Journal of Nursing Practice* 2007, 13(4):243-253 [51].UK | Method: Quantitative secondary analysis of PACT data (July 2004-June 2005) | UK Nurse prescribing from (NPF) & NPEF | Analysis found little variation in the number of items prescribed on a month-on-month basis by both nurses and GPs. A total of 37,467 items per month were prescribed by nurses; in comparison the volume of items prescribed by GPs was substantially greater with a mean of 913181.0 items being prescribed per month. Study found wide regional variations, for example, in North East of England nurses prescribed a total of 2422 items; in contrast North East London 91 nurses prescribed a total of 439 items. Community nurses employed by the Primary Care Trust prescribed the greater majority of laxative items (84.9%) and prescribed five times more (5281) items than general practice-employed nurses (960). | The generalisability of the study findings to prescribing patterns in relation to other clinical situations is limited without supporting data. | The aim was to explore prescribing patterns of primary care nurses across England through prescriptions for laxatives.  No stated research question.  The analysis methods are not described in detail. |
| Kimmer E, Christian A: A review of the usefulness and efficacy of independent nurse prescribing. *Nurse Prescribing* 2005, 3(1):39-42 [58]. UK | Method: Quantitative observational survey.  Data were collected over a period of 6 weeks from January to March 2004 using data collection form. In total two nurses saw 744 patients during 64 sessions. | UK Nurse Prescribing from NPEF | Study reported on the perceived usefulness and efficiency of NP as improvement of patients’ access to medicines and the nurses complete episodes of care in 65% of patients presenting in the same-day appointments by a combination of advice and nurse prescriptions. | None stated. | The question is not clearly stated and the method was not clearly described. It could be described as an observational study or a consultation review as the practice nurses have designed a data collection form to complete recording the reason for patient consultation and treatment given. |
| Boonstra E, Lindbaek M, Khulumani P, Ngome E, Fugelli P: Adherence to treatment guidelines in primary health care facilities in Botswana. *Tropical Medicine & International Health: TM & IH* 2002, 7(2):178-186 [46]. Botswana | Method: mixed methods- questionnaire survey & participants observation  Sample: 2994 consultations in three of 22 administrative districts at 30 primary health care facilities | Botswana Nurse Prescribing | The average number of drugs prescribed per patient was 2.3. Antibiotics were prescribed in 27% of all encounters. Full adherence (complete adherence to national recommended treatment guidelines) was found in 44%, acceptable compliance in 20%, `acceptable, but one or more useless, but not dangerous, drugs' in 33% and `insufficient or dangerous treatment' in 3% of the consultations. | None stated. | The question is clearly focussed but there is no detail on how nurses were selected to take part or whether they are representative. Methods are clearly reported, participatory observation by a nurse/midwife from the research team. |
| Shum C, Humphreys A, Wheeler D: Nurse management of patients with minor illnesses in general practice: multicentre randomised controlled trial. *British Medical Journal* 2000, 320(7241):1038-1043 [52].UK | Method: Quantitative Randomised Control Trial  Sample: 1815 patients allocated to treatment  Setting: 5 general practices | UK Nurse prescribing type not specified | This study found that nurses and doctors wrote prescriptions for a similar proportion of patients (nurses 481/736 (65.4%) v doctors 518/816 (63.5%). However, nurses reported giving more advice on self medication and general self management than doctors. | This trial did not examine the content of the consultation in detail and the study did not have enough power to detect difference in rare outcomes. | A clear research objective: To assess the acceptability and safety of a minor illness service led by practice nurses in general practice. |
| Hamric AB, Worley D, Lindebak S, Jaubert S: Outcomes associated with advanced nursing practice prescriptive authority. *Journal of the American Academy of Nurse Practitioners* 1998, 10(3):113-118 [53]. USA | Method: Quantitative multi-methods survey  Thirty-three advanced practice nurses (APNs) in 25 different primary care sites in one state participated in a study of the safety and effectiveness of APN prescriptive authority. Data were analyzed on 1,708 patients seen during a 2-month period. | USA Collaborative Practice Nurse Prescribing | The study demonstrated that overall patient outcomes were positive; over half (59%) of the patients improved and 76% of the patients either improved or stabilised in response to APN treatment. Patient assessments of their outcomes were similarly positive. Patients experienced low waiting times (63% waited 15 minutes or less) and were highly satisfied with APN care. | The lack of verifiable outcomes for many of the patients seen by the APN was a weakness of this study. | Method not described in detail although objectives are clearly reported. The study is described as a demonstration project and appears to be a multi-method evaluation study involving review of consultation documentation with assessment of appropriateness of diagnosis/treatment by collaborating physician and patient satisfaction survey. |
| Stein CM, Todd WT, Parirenyatwa D, Chakonda J, Dizwani AG: A survey of antibiotic use in Harare primary care clinics. *The Journal Of Antimicrobial Chemotherapy* 1984, 14(2):149-156 [54]. Zimbabwe | Method: Quantitative drug survey  Sample: 1000 patients drug survey | Zimbabwe Nurse prescribing | This survey of antibiotic prescribing by nurses found that 543 of 1000 patients were treated with antibiotics. Penicillin was the only antibiotic used in 444 patients. Respiratory and soft tissue infections were the commonest reasons for prescribing an antibiotic. Because of the design of the survey, a full audit of antibiotic use was not possible but antibiotic use was deemed inappropriate in 12.3% of patients. | The study examined the use of an antibiotic for a stated or implied diagnosis. However, no attempt was made to verify this diagnosis. | Question is not clearly focused and method is also not specified although appears to be a medical records review/survey. |
